# A Recoverable Curved Multilayer Cornea-on-a-Chip with DRGO-Derived Neurites

**DOI:** 10.3390/biomedicines14102242

**Published:** 2026-10-03

**Authors:** Xi Wang, Jianing Gu, Chengcheng Ding, Jiansu Chen, Zekai Cui

**Affiliations:** 1 Aier Academy of Ophthalmology, Central South University, Changsha 410083, China; 248211159@csu.edu.cn (X.W.); chenjiansu2000@163.com (J.C.); 2 Changsha Aier Eye Hospital, Changsha 410015, China; gujianing@aierchina.com (J.G.); dingchengcheng@aierchina.com (C.D.); 3 Aier Eye Institute, Changsha 410083, China

**Keywords:** cornea-on-a-chip, curved corneal model, air-liquid interface, dorsal root ganglion organoid, corneal innervation

## Abstract

**Background:** In vitro corneal models that combine curved geometry, multilayer tissue organization, neural components, air–liquid interface (ALI) culture, and perfusion remain limited. **Methods:** We developed a recoverable curved multilayer cornea-on-a-chip (CMCoC) using a two-component polydimethylsiloxane device with a 7.8-mm-radius dome. The construct comprised an HCE-T-derived epithelial layer, human corneal stromal cells embedded in plastically compressed type I collagen, an HCEC-B4G12-containing endothelial compartment, and circumferential human induced pluripotent stem cell-derived dorsal root ganglion organoids (DRGOs). After assembly, constructs were perfused for 7 days to permit peripheral neurite extension, followed by epithelial seeding and 14 days of ALI culture with continued basal perfusion. **Results:** Retrieved tissues showed a visible curved profile. Immunofluorescence demonstrated the intended epithelial–stromal–endothelial organization, a multilayered epithelium, and extensive βIII-tubulin-positive DRGO-derived neurites associated with the epithelial compartment. The CMCoC had a thinner epithelial compartment than the mouse corneal reference and a higher βIII-tubulin-positive area fraction. The other three neural morphometric comparisons were not statistically significant after adjustment. **Conclusions:** The CMCoC combines recoverable curved tissue architecture, multicellular organization, and DRGO-derived neurites, providing a basis for future studies of corneal tissue interactions.

## 1. Introduction

The cornea is a transparent, avascular, and highly organized tissue that provides the principal refractive interface of the eye while protecting the intraocular environment [1]. Its properties arise from the coordinated organization of a stratified epithelium, a collagen-rich stroma containing keratocytes, and a posterior endothelial monolayer [2]. Stromal collagen architecture is central to transparency [3], while the endothelium contributes to regulation of stromal hydration through ion and fluid transport mechanisms [4]. The cornea is also densely innervated. Corneal nerves detect mechanical, thermal, and chemical stimuli and participate in epithelial maintenance, tear reflexes, wound healing, and ocular-surface homeostasis [5,6]. Experimental models that integrate cellular composition with multilayer organization, curved geometry, neural-epithelial relationships, and distinct apical and basal environments are therefore relevant to studies of corneal homeostasis, injury, and treatment response.

Corneal biology has been investigated using two-dimensional cultures, collagen-based constructs, tissue-engineered corneas, explants, animal models, and microfluidic platforms. Cornea-on-a-chip systems complement established models by permitting controlled compartmentalization, fluid delivery, co-culture, and dynamic exposure [7,8]. Existing platforms have been used to investigate epithelial wound healing [9], dry-eye-associated epithelial changes under ALI conditions [10], bacterial keratitis and antimicrobial responses [11], and multiscale interactions at the human ocular surface [12]. Dynamic in vitro systems have also been developed to model blinking and ophthalmic formulation behavior [13]. These studies establish the utility of application-specific corneal microphysiological models. Relatively few systems, however, integrate curved geometry, epithelial, stromal, and endothelial cellular compartments, peripheral neural elements, an apical ALI, basal perfusion, and tissue recovery in one platform.

A central anterior human corneal radius near 7.8 mm provides a representative geometric reference for model design, although curvature varies among individuals and across the corneal surface [14]. Recent organ-chip evidence further indicates that substrate curvature can influence corneal stromal-cell alignment and phenotype [15]. Multilayer innervated corneal constructs and compartmentalized neural microfluidic platforms establish the feasibility of integrating organized corneal tissue with peripheral neural elements [16,17], while quantitative syntheses emphasize that corneal nerve measurements depend on biological and imaging context [18]. Advances in directing human pluripotent stem cells toward sensory neuronal lineages, including dorsal root ganglion organoid-derived populations, provide additional neural sources for engineered systems [19,20,21,22]. Together, these developments motivated a model that integrates corneal curvature, cellular layering, controlled exposure compartments, and peripheral neurite extension.

On this basis, we designed a curved multilayer cornea-on-a-chip (CMCoC) that combines a 7.8-mm-radius culture surface, epithelial, stromal, and endothelial cellular compartments, circumferential human induced pluripotent stem cell-derived dorsal root ganglion organoids (DRGOs), an apical ALI, and basal perfusion. The apical configuration reflects the importance of tear-film exposure at the ocular surface [23], while the use of polydimethylsiloxane (PDMS) requires recognition of its potential absorption of small molecules in future drug studies [24]. More broadly, organ-chip technologies can connect tissue engineering with controlled microfabrication and may support disease modeling and drug development [25,26,27]. After application-specific validation, such systems may also contribute to Reduction and selected forms of Replacement within the 3R framework [28]. In this study, we evaluated device assembly, tissue recovery and post-retrieval morphology, the intended epithelial-stromal-endothelial arrangement, epithelial thickness, and the distribution and morphometry of βIII-tubulin-positive DRGO-derived neurites. Mouse corneas were used as anatomical and morphometric references. This study focuses on the assembly and structural characterization of an integrated, recoverable corneal model as a basis for future investigations of corneal tissue interactions.

## 2. Materials and Methods

### 2.1. Ethics Statement and Biological Materials

Human corneal stromal cells (CSCs) were isolated from stromal lenticules obtained during small-incision lenticule extraction (SMILE) surgery. The use of lenticule tissue samples was approved by the Ethics Committee of Aier Eye Hospital, Changsha, Hunan, China (No. 2023-KYPJ009) and adhered to the principles of the Declaration of Helsinki. Written informed consent was obtained from all patients. Five eight-week-old male C57BL/6 mice were used, and both corneas were collected from each animal. One cornea from each mouse was used for cross-sectional immunofluorescence analysis, and the contralateral cornea was allocated to whole-mount preparation. All experimental procedures were conducted in strict accordance with the ARVO Statement for the Use of Animals in Ophthalmic and Vision Research and were approved by the Animal Welfare and Ethics Committee of the Aier Eye Institute of Hunan Province (Approval No. AEI20230068).

### 2.2. Design and Fabrication of the CMCoC

**Figure 1:**
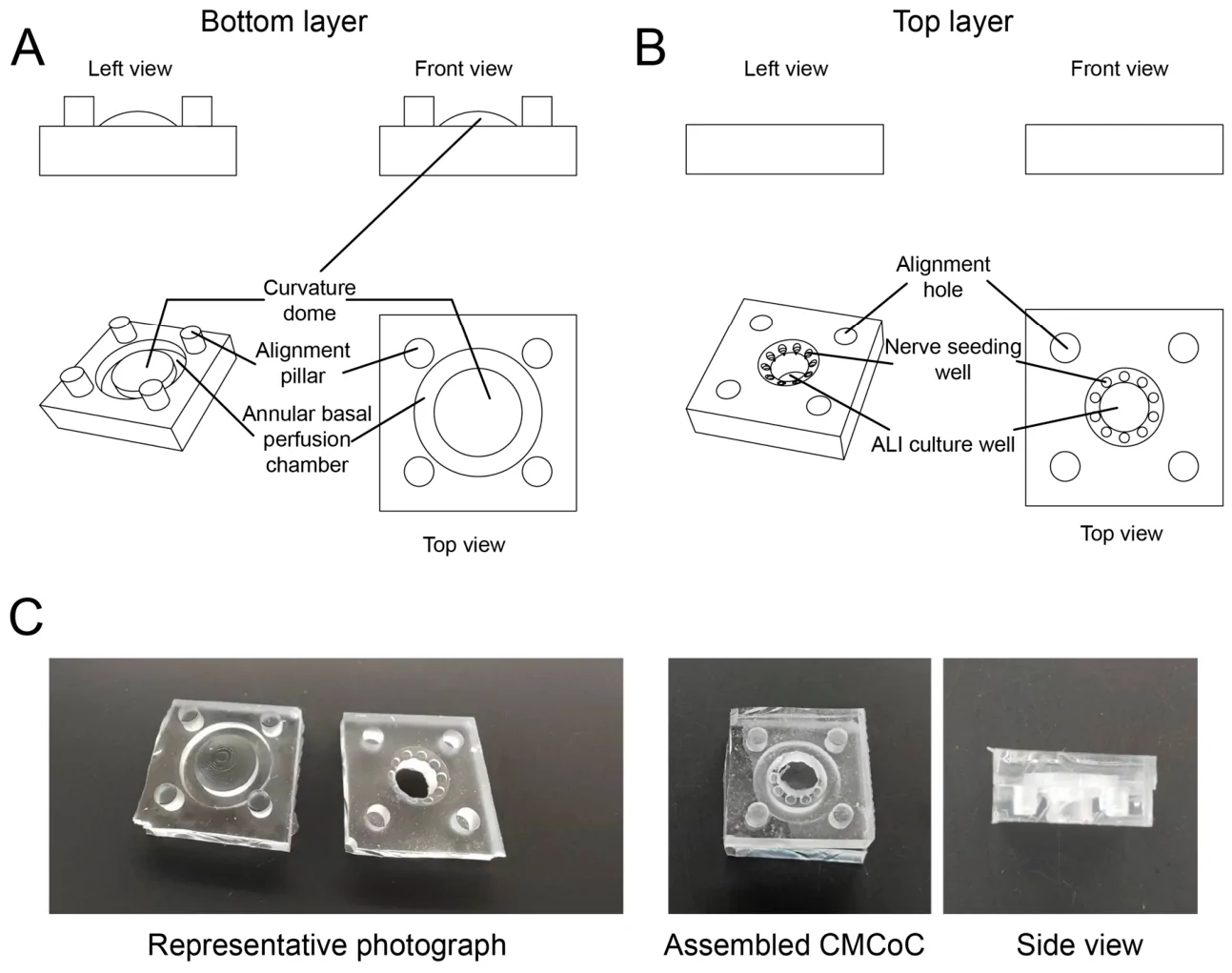
Design and fabrication of the curved multilayer cornea-on-a-chip. (**A**) Schematic views of the lower PDMS component, showing the 7.8-mm-radius curvature dome, alignment pillars, and the 2-mm-deep annular basal perfusion chamber. The annular chamber is the structure labeled “Annular basal perfusion chamber” in the schematic and is distinct from the inlet and outlet channels connected to it. (**B**) Schematic views of the upper PDMS component, showing alignment holes, the central ALI culture well, and circumferential DRGO-seeding wells. (**C**) Representative photographs of the separated PDMS components, the assembled CMCoC, and its side view.

The CMCoC consisted of complementary upper and lower PDMS components. The lower component contained a central curvature dome with a radius of 7.8 mm, designed to approximate a representative central anterior human corneal radius, together with alignment pillars and a 2-mm-deep annular basal perfusion chamber. This annular chamber is the structure labeled “Annular basal perfusion chamber” in the Figure 1 schematic and was connected to distinct inlet and outlet channels. The upper component contained corresponding alignment holes, a central ALI culture well, and circumferential wells for DRGO placement.

Master molds for the upper and lower components were fabricated using an LCD vat-photopolymerization 3D printer equipped with a 405-nm light source (Photon Mono X, Anycubic Technology Co., Ltd., Shenzhen, China). SUNLU Standard yellow-green acrylate-based photocurable resin (SUNLU Technology Co., Ltd., Dongguan, China) was used. The XY resolution and Z-layer thickness were both 50 μm. Normal-layer and bottom-layer exposure times were 2.5 and 35 s, respectively. The STL files for the 3D-printed upper and lower molds are provided in the Supplementary Materials.

Sylgard 184 PDMS prepolymer and curing agent (Dow Corning, Midland, MI, USA) were mixed at a 10:1 ratio, degassed, poured into the corresponding molds, and cured at 55 °C for 4 h. After demolding, inlet and outlet ports were prepared, and the PDMS components were sterilized by autoclaving at 121 °C. The upper and lower PDMS components were aligned and held in contact by mechanical engagement of the alignment pillars and corresponding holes, without permanent bonding. This assembly allowed the components to be separated for tissue retrieval after culture.

### 2.3. Cell Culture and DRGO Differentiation

SMILE-derived stromal lenticules were digested overnight at 37 °C using 0.1% (*w*/*v*) type I collagenase (Sigma-Aldrich, St. Louis, MO, USA). Isolated CSCs were maintained in DMEM/F12 supplemented with 5% fetal bovine serum and 1% penicillin-streptomycin (Gibco, Grand Island, NY, USA). Cells were passaged at a ratio of 1:3 every 3 days using 0.25% trypsin. DMEM/F12, fetal bovine serum, and trypsin were obtained from Thermo Fisher Scientific (Waltham, MA, USA).

Corneal endothelial tissue-engineering studies provided methodological context for establishing a cultured basal cellular layer [29]. HCE-T human corneal epithelial cells (CEpCs) and HCEC-B4G12 human corneal endothelial cells (CEnCs) were purchased from Shanghai Hongshun Biotechnology Co., Ltd. (Shanghai, China). Both cell lines were maintained in DMEM/F12 containing 10% fetal bovine serum and 1% penicillin-streptomycin at 37 °C under 5% CO_2_ and were passaged at a ratio of 1:3 every 3 days using 0.25% trypsin.

A urine-derived human induced pluripotent stem cell line was purchased from Cellapy Biotechnology (Beijing, China). Cells were maintained on cultureware coated with Matrigel (Corning, Corning, NY, USA) in mTeSR Plus medium (STEMCELL Technologies, Vancouver, BC, Canada) and passaged at a ratio of 1:8 every 3–4 days using 0.5 mM EDTA. DRGOs were generated according to the published protocol [30], as summarized in Figure S1A. Cells were seeded on day −2 in knockout serum replacement (KSR; Thermo Fisher Scientific, Waltham, MA, USA)-based medium, followed by a progressive transition to N2 medium between days 4 and 10. Y-27632 (MedChemExpress, Monmouth Junction, NJ, USA) was included during days −2 to −1, and SB431542 (MedChemExpress, Monmouth Junction, NJ, USA) and LDN193189 (MedChemExpress, Monmouth Junction, NJ, USA) were included from day 0 to day 10. CHIR99021, SU5402, and DAPT (MedChemExpress, Monmouth Junction, NJ, USA) were added during days 4–10. From day 10, supplementation included brain-derived neurotrophic factor, glial cell-derived neurotrophic factor, nerve growth factor, neurotrophin-3 (PeproTech, Cranbury, NJ, USA), and ascorbic acid (Sigma-Aldrich, St. Louis, MO, USA). DRGOs were collected between days 16 and 26, and 10 organoids were placed in each CMCoC. Immunophenotypic characterization was performed on day-26 DRGOs before chip incorporation (Figure S1B).

CSCs isolated from SMILE-derived lenticules were used as a pooled cell preparation. All DRGOs used for chip assembly originated from a single differentiation batch. The five CMCoCs were prepared simultaneously in one experimental run using the pooled CSC preparation and DRGOs from this batch, with ten DRGOs placed in each device.

### 2.4. Preparation of CSC-Laden Collagen Membranes

CSC-laden collagen membranes were prepared by plastic compression as previously described [30,31,32,33]. A total of 4 × 10^6^ passage-3 CSCs were mixed with 9 mL of precooled 0.5% (*w*/*v*; 5 mg/mL) bovine tendon-derived type I collagen (Guangzhou Trauer Biotechnology, Guangzhou, China) and 1 mL of 10× DMEM (HyClone Laboratories, Logan, UT, USA). One milliliter of the mixture, containing 4 × 10^5^ CSCs, was added to each well and allowed to gel at 37 °C for 30 min.

A 500-mesh nylon ring was placed on each side of the collagen hydrogel to support the construct during transfer and inversion. Absorbent filter paper was positioned around the hydrogel, followed by a glass plate and a 100-g load. The hydrogel was compressed for 10–20 min to produce a thin CSC-laden collagen membrane.

### 2.5. Sequential CMCoC Assembly and Perfusion Culture

The surface intended to form the basal HCEC-B4G12 CEnC-containing layer was coated with laminin-521 (Thermo Fisher Scientific, Waltham, MA, USA) at 0.75 µg/cm^2^ and type IV collagen (Sigma-Aldrich, St. Louis, MO, USA) at 5 µg/cm^2^ for 1 h at 37 °C. HCEC-B4G12 CEnCs were seeded at 2 × 10^6^ cells/mL and allowed to adhere for 1 h at 37 °C under 5% CO_2_. The endothelial cell-seeded membrane was subsequently cultured for 2 days.

The nylon mesh supports were removed before assembly. The collagen membrane was positioned over the curvature dome with the HCEC-B4G12-containing surface facing the basal perfusion chamber. DRGOs were placed in the circumferential wells, after which the upper and lower PDMS components were assembled.

The basal chamber was connected to an ISMATEC Reglo ICC peristaltic pump (Cole-Parmer, Vernon Hills, IL, USA) through silicone tubing with an internal diameter of 0.13 mm. Before perfusion, the tubing was primed with culture medium. A single-pass configuration was used, in which medium was supplied from a reservoir containing 50 mL of culture medium, passed through the chip, and collected in a separate waste bottle. The medium in the supply reservoir was replaced every 5 days. DRGO differentiation medium was continuously perfused at 2.0 μL/min. This rate was selected with reference to physiological human aqueous humor flow, for which daytime mean rates of approximately 2–3 μL/min have been reported [34,35]. The nominal daily flow throughput was 2.88 mL. The assembled CMCoC was maintained under basal perfusion for 7 days before epithelial seeding.

A 100-µL aliquot of HCE-T CEpC suspension at 3 × 10^6^ cells/mL was applied to the apical surface. After 1 day of attachment, the apical medium was removed to establish an air–liquid interface (ALI). The HCE-T CEpC-derived epithelial layer was oriented toward the apical air interface, whereas the HCEC-B4G12 CEnC-containing layer faced the basal perfusion chamber. During co-culture, constructs were maintained in CSC culture medium supplemented with 10 ng/mL nerve growth factor (NGF). Basal single-pass perfusion was maintained at 2.0 μL/min throughout ALI culture. After ALI establishment, no medium was added to the apical surface. Constructs were maintained for a further 14 days at 37 °C in a humidified incubator with 5% CO_2_. At the end of culture, the two PDMS components were separated and the curved tissue was retrieved.

### 2.6. Immunofluorescence Staining and Microscopy

CMCoC constructs and mouse corneas were radially cut and flattened for whole-mount imaging. Each CMCoC was divided into four connected petals to facilitate flattening. Z-stacks from both tissue types were processed as maximum-intensity projections for neural morphometric analysis. Samples were fixed in 4% paraformaldehyde for 15 min, followed by permeabilization and blocking for 1 h in PBS containing 3% bovine serum albumin and 1% Triton X-100. Primary antibodies against βIII-tubulin, ZO-1, and ALDH3A1 were used at a dilution of 1:200 (Proteintech, Rosemont, IL, USA). Additional antibodies for DRGO characterization included mouse anti-Brn3 (1:500; Santa Cruz Biotechnology, Dallas, TX, USA), mouse anti-Bassoon (Bsn; 1:200; Santa Cruz Biotechnology, Dallas, TX, USA), rabbit polyclonal anti-S100B (1:500; Bioss, Beijing, China), rabbit polyclonal anti-GABA transporter 1 (GAT1; 1:500; Synaptic Systems, Göttingen, Germany), rabbit polyclonal anti-Synaptogyrin 1 (1:500; Synaptic Systems, Göttingen, Germany), and rabbit polyclonal anti-VGLUT1 (1:200; Synaptic Systems, Göttingen, Germany). Corneal compartment characterization additionally used mouse monoclonal anti-CK3 (1:200; Abcam, Cambridge, UK), rabbit polyclonal anti-Na^+^/K^+^-ATPase (1:200; Proteintech, Rosemont, IL, USA), and rabbit polyclonal anti-α-SMA (1:500; Proteintech, Rosemont, IL, USA). Samples were incubated overnight with the appropriate primary antibodies for each staining panel. After washing with PBS, samples were incubated for 1 h with species-matched goat anti-mouse or goat anti-rabbit IgG secondary antibodies conjugated to Alexa Fluor 488 or Alexa Fluor 594 (1:1000; Invitrogen, Thermo Fisher Scientific, Carlsbad, CA, USA). F-actin was labeled with fluorescent phalloidin (1:1000; Beyotime, Shanghai, China), and nuclei were counterstained with DAPI. ALDH3A1 immunoreactivity was assessed across the epithelial and stromal compartments. Additional immunofluorescence characterized day-26 DRGOs before chip incorporation and the epithelial and endothelial compartments at the chip-culture endpoint. The DRGO panel included βIII-tubulin, Brn3, S100B, Bassoon, GAT1, Synaptogyrin 1, and VGLUT1. CK3 and Na^+^/K^+^-ATPase were examined in the endpoint HCE-T and B4G12 compartments, respectively. Cross-sectional tissue analysis additionally included α-SMA staining. Whole-mount confocal images and their optical and orthogonal views were acquired using a Zeiss LSM 880 microscope (Carl Zeiss Microscopy, Jena, Germany).

### 2.7. Morphometric and Statistical Analyses

Epithelial thickness was measured in Fiji (ImageJ2 version 2.1.0; ImageJ version 1.53i) on cross-sectional immunofluorescence images by drawing measurement lines perpendicular to the local tissue surface. Multiple measurements from each specimen were averaged, and each independent CMCoC or mouse cornea was treated as one biological replicate (*n* = 5 per group). Neural morphometry was analyzed in four CMCoCs and four mouse corneal whole mounts (*n* = 4 per group). Maximum-intensity projections of βIII-tubulin fluorescence were analyzed in Fiji (ImageJ). Radial incisions, tissue-free areas, DRGO bodies, circular defects, and stitching artifacts were excluded from the analyzed tissue regions. Automatic thresholding was used to generate binary masks of βIII-tubulin-positive structures. Area fraction was calculated as the positive area divided by the analyzed tissue area. Binary masks were skeletonized to quantify total structure length, branch points, and endpoints in the projected networks. Structure length density was calculated as total skeletonized length divided by the analyzed tissue area and expressed as mm/mm^2^. Branch-point and endpoint densities were calculated by dividing their respective counts by the analyzed tissue area and expressed as mm^−2^. Image analysis was performed with knowledge of group identity. Original data tables and plots were prepared using GraphPad Prism 8.0. Statistical reanalysis was performed in Python 3.12.14 using SciPy 1.18.1. Data are presented as mean ± SD. Between-group comparisons were performed using two-sided Welch’s *t*-tests. Holm correction was applied across the four neural morphometric outcomes, with adjusted *p* < 0.05 used for those comparisons. Epithelial thickness was analyzed separately. Mean differences are reported as CMCoC minus mouse cornea with unadjusted 95% confidence intervals. Summary statistics and individual source values are provided in Table S1 and Table S2, respectively.

## 3. Results

### 3.1. The Two-Component CMCoC Provides a Curved and Compartmentalized Corneal Culture Interface

The CMCoC was designed as a two-component PDMS device that spatially organized the corneal construct and peripheral neural components (Figure 1). The lower component contained a central dome with a radius of 7.8 mm, designed to approximate a representative central anterior human corneal radius, together with a surrounding 2-mm-deep annular basal perfusion chamber. This chamber corresponds to the structure labeled “Annular basal perfusion chamber” in the schematic and was connected to separate inlet and outlet channels. Four alignment pillars facilitated positioning of the device components (Figure 1A).

The upper component contained a central ALI culture well aligned with the dome and multiple circumferential nerve-seeding wells for DRGO placement. Alignment holes matched the pillars in the lower component and stabilized the tissue construct during assembly and culture (Figure 1B). Photographs confirmed that the complementary PDMS components could be assembled into a compact device with a central curved culture compartment (Figure 1C). These structural features established an apical ALI compartment, basal perfusion access, and spatially separated sites for peripheral DRGO placement.

### 3.2. Sequential Bioassembly Generates a Harvestable Curved Corneal Construct

**Figure 2:**
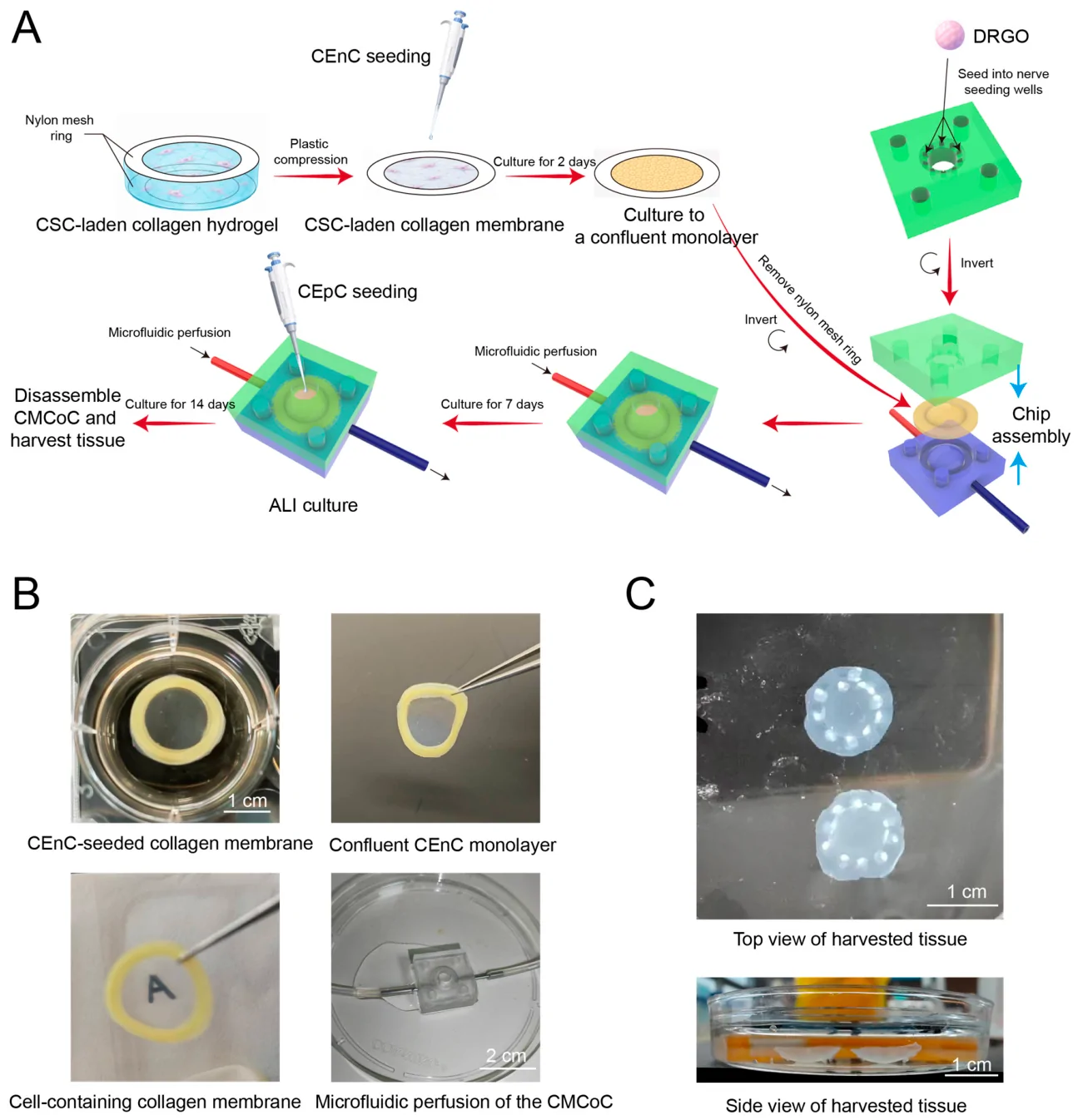
Sequential assembly, perfusion culture, and recovery of the CMCoC tissue. (**A**) CSCs were embedded in type I collagen, supported between nylon mesh rings, and subjected to plastic compression. HCEC-B4G12 CEnCs were seeded on the surface intended to face the basal perfusion chamber and cultured for 2 days. After removal of the nylon supports, the membrane and DRGOs were assembled in the device and perfused for 7 days to permit peripheral neurite extension. HCE-T CEpCs were then seeded apically, allowed to attach for 1 day, and cultured for a further 14 days under ALI conditions with basal perfusion. (**B**) Representative photographs showing the macroscopic appearance of the endothelial-cell-seeded collagen membrane and the assembled culture system under perfusion. (**C**) Top and side views of recovered tissue showing its circular outline and visible curved profile.

A sequential culture strategy was used to integrate CSCs, HCEC-B4G12 cells, HCE-T cells, and DRGOs within the CMCoC (Figure 2A). SMILE-derived CSCs were embedded in a collagen hydrogel, and nylon mesh rings placed on both sides supported handling during transfer and inversion. Plastic compression converted the hydrogel into a thin CSC-laden collagen membrane.

HCEC-B4G12 cells were seeded onto one surface of the compressed collagen membrane and cultured for 2 days. After removal of the nylon supports, the endothelial cell-seeded membrane was positioned over the curvature dome, DRGOs were placed in the circumferential wells, and the two PDMS components were assembled. The construct was perfused for 7 days before epithelial seeding to permit DRGO-derived neurites to extend from the peripheral wells toward the central tissue. HCE-T cells were subsequently seeded onto the apical surface, followed by 1 day of attachment and a further 14 days of ALI culture with basal perfusion. Representative photographs documented the macroscopic appearance of the endothelial-cell-seeded collagen membrane and the assembled CMCoC under perfusion (Figure 2B).

At the end of culture, the device could be disassembled and the tissue retrieved. Harvested constructs had a circular outline with peripheral DRGO-associated structures, and side-view imaging showed a visible curved profile after retrieval (Figure 2C). The procedure therefore generated a manipulable and harvestable curved tissue for subsequent structural assessment.

### 3.3. CMCoC Forms a Multilayered Tissue with the Intended Corneal Cellular Arrangement and Integrated Neurite Extension

**Figure 3:**
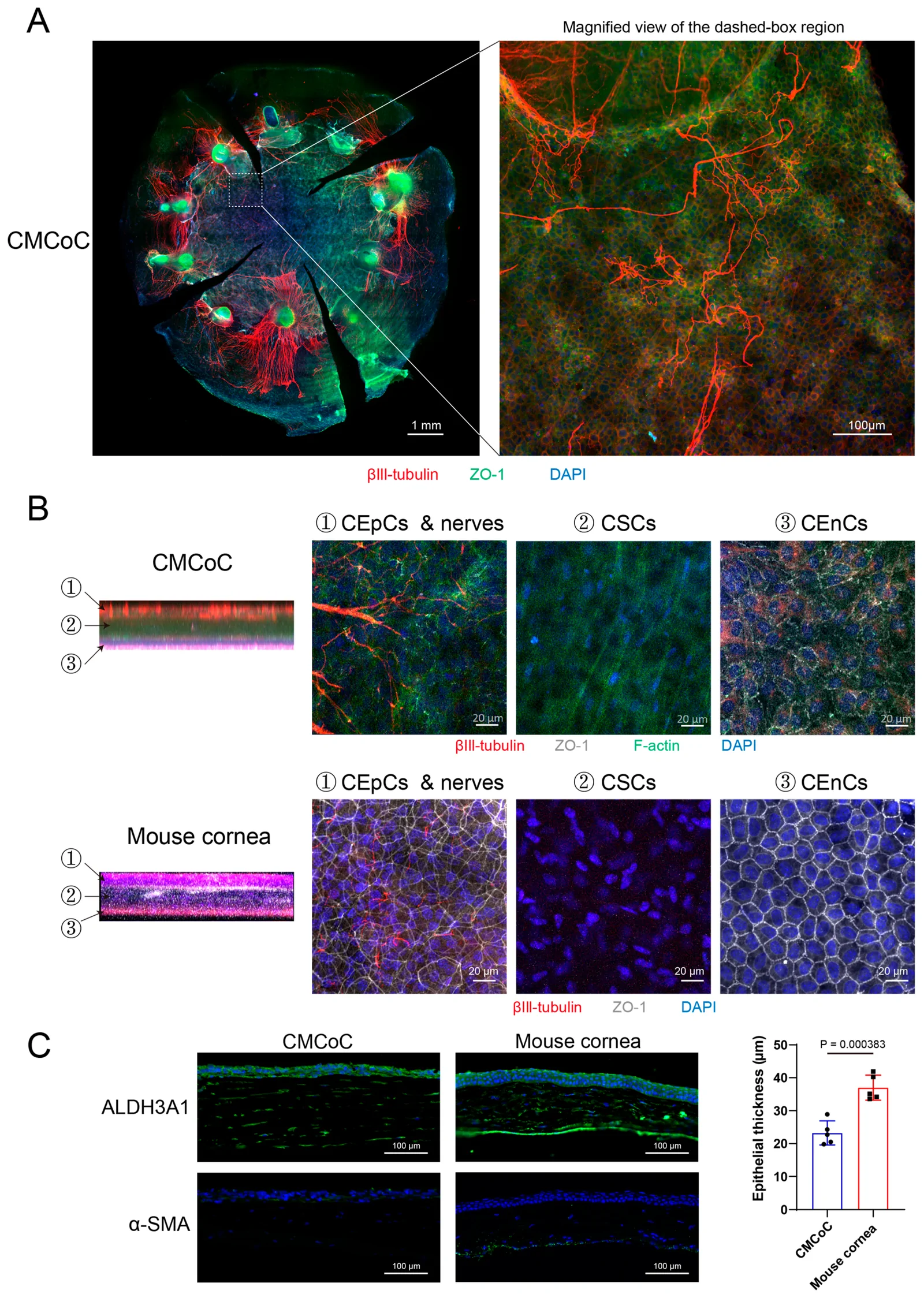
Cellular organization, neurite-epithelial association, and epithelial thickness in the CMCoC. (**A**) Whole-mount and higher-magnification immunofluorescence images of the CMCoC showing βIII-tubulin-positive DRGO-derived neurites, ZO-1-associated epithelial organization, and DAPI-labeled nuclei. Radial incisions were introduced to flatten the curved tissue. The right image shows a magnified view of the dashed-box region in the left overview. Scale bars: 1 mm and 100 µm, respectively. (**B**) Orthogonal projections and representative images of the intended epithelial, stromal, and endothelial cellular compartments in the CMCoC and corresponding regions in mouse corneal reference tissue. Markers shown include βIII-tubulin, ZO-1, F-actin, and DAPI. Scale bars: 20 µm in cellular images. (**C**) Cross-sectional immunofluorescence images showing ALDH3A1 and α-SMA labeling and DAPI-counterstained nuclei in CMCoC and mouse corneal reference tissues. ALDH3A1 immunoreactivity is visible in epithelial and stromal regions. Scale bars: 100 μm. Epithelial-thickness measurements were averaged within each specimen before analysis. Individual specimen values and mean ± SD are shown (five specimens per group). The CMCoC–mouse mean difference was −13.78 μm (95% CI, −19.21 to −8.35 μm; two-sided Welch’s *t*-test, *p* = 0.000383).

Whole-mount immunofluorescence imaging was used to examine epithelial and neural organization after tissue recovery. Radial incisions permitted flattening of the curved tissue for imaging. βIII-tubulin-positive neurites extended from the circumferential DRGOs into the construct and covered a substantial portion of the tissue (Figure 3A). Higher-magnification imaging showed branched neurites distributed across and between ZO-1-positive epithelial cells, demonstrating close spatial association between the DRGO-derived neurite network and the epithelial compartment.

Orthogonal projections showed distinct cellular distributions across the tissue thickness (Figure 3B). The apical compartment contained the HCE-T-derived epithelial layer and βIII-tubulin-positive neurites. CSCs were distributed within the collagen stromal layer, whereas HCEC-B4G12 cells occupied the opposite surface. ZO-1 and F-actin delineated cellular organization. The observed distribution was consistent with the intended epithelial-stromal-endothelial arrangement.

Representative cross-sectional immunofluorescence images showed a multilayered epithelial compartment overlying the CSC-containing collagen matrix (Figure 3C). ALDH3A1 immunoreactivity was observed in both the epithelial and stromal compartments. Epithelial-thickness measurements were averaged within each specimen before analysis. Individual specimen values and mean ± SD are shown (five specimens per group). Epithelial thickness was 23.24 ± 3.63 μm in CMCoC specimens and 37.02 ± 3.80 μm in mouse corneas. The mean difference was −13.78 μm (95% CI, −19.21 to −8.35 μm; two-sided Welch’s *t*-test, *p* = 0.000383), while the engineered epithelium remained multilayered rather than forming a single-cell monolayer.

Immunofluorescence of day-26 DRGOs showed βIII-tubulin-positive neurites together with labeling for Brn3, S100B, Bassoon, GAT1, Synaptogyrin 1, and VGLUT1 before chip incorporation (Figure S1B). At the chip-culture endpoint, CK3 was not readily detectable in the HCE-T compartment, whereas Na^+^/K^+^-ATPase immunoreactivity was detected in the B4G12 compartment (Figure S2). No conspicuous α-SMA immunoreactivity was observed in the examined stromal regions (Figure 3C).

### 3.4. CMCoC Develops an Extensive and Highly Branched Neurite Network

**Figure 4:**
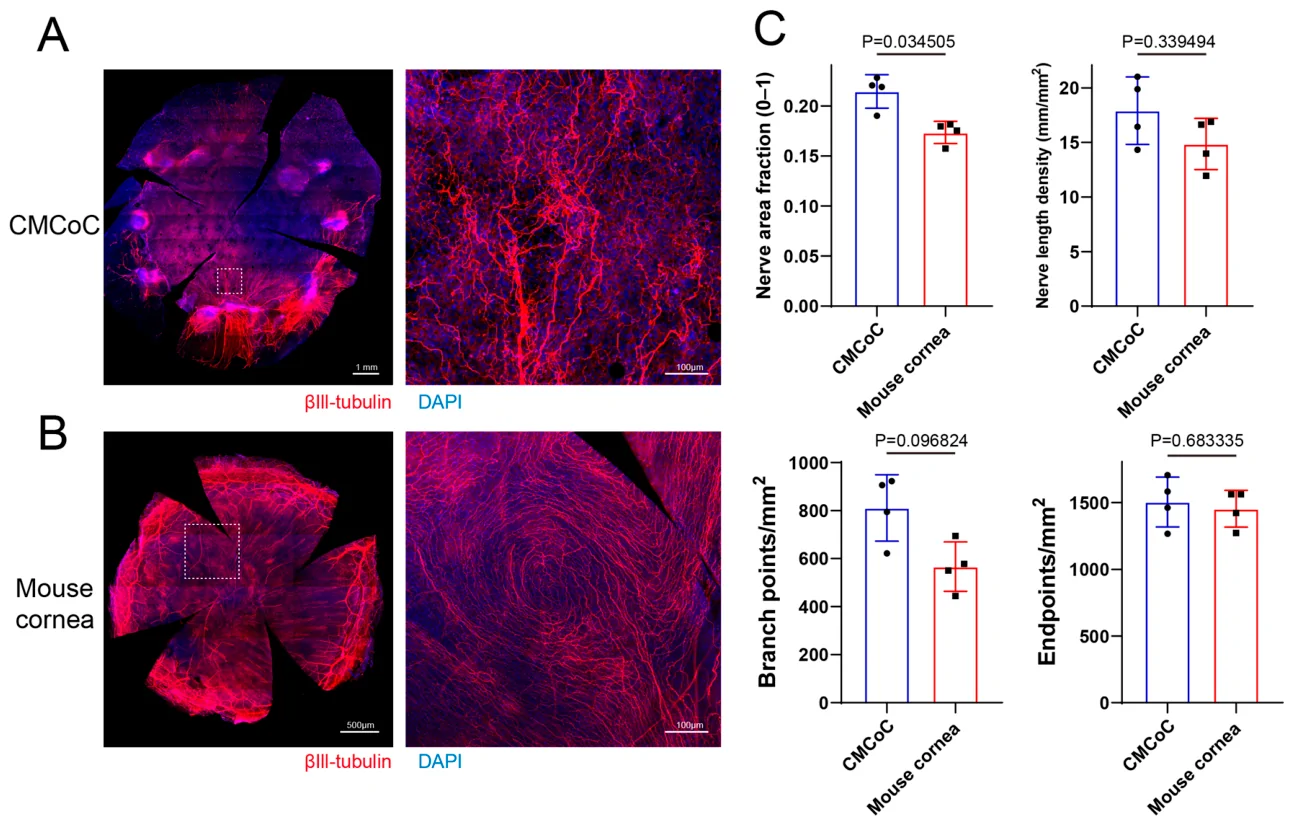
Morphometric characterization of the CMCoC neurite network. (**A**) Whole-mount and higher-magnification βIII-tubulin immunofluorescence images showing DRGO-derived neurites in the CMCoC; nuclei were counterstained with DAPI. Scale bars: 1 mm and 100 µm. (**B**) Whole-mount and higher-magnification images of βIII-tubulin-positive corneal nerves in mouse reference tissue. Scale bars: 500 µm and 100 µm. (**C**) Quantification of neurite area fraction (0–1), structure length density (mm/mm^2^), branch-point density (mm^−2^), and endpoint density (mm^−2^). Each point represents one specimen-level value obtained by averaging measurements within that specimen (four specimens per group); bars show mean ± SD. Comparisons used two-sided Welch’s *t*-tests with Holm correction across the four outcomes. Adjusted *p* values are reported in the panel; unadjusted *p* values and estimated differences with unadjusted 95% confidence intervals are provided in Table S1.

Whole-mount βIII-tubulin staining was used to examine the organization of DRGO-derived neurites in the CMCoC and corneal nerves in the mouse reference (Figure 4). In the CMCoC, neurite bundles emerged from peripherally positioned DRGOs and divided into finer processes toward the central tissue. Higher-magnification imaging showed an interconnected network of thin and highly branched neurites throughout the epithelial compartment (Figure 4A). Mouse corneas showed a radial organization of larger nerve trunks and a dense epithelial nerve pattern toward the central cornea (Figure 4B).

The βIII-tubulin-positive area fraction was higher in CMCoC than in the mouse corneal reference after correction for the four neural comparisons (Holm-adjusted *p* = 0.034505). Mean branch-point density was also higher in CMCoC, but the adjusted comparison did not reach statistical significance (*p* = 0.096824). The adjusted *p* values for structure length density and endpoint density were 0.339494 and 0.683335, respectively. Table S1 reports the four outcome summaries, estimated differences, confidence intervals, and unadjusted and adjusted *p* values. These measurements characterize the distribution and branching of βIII-tubulin-positive structures in the analyzed tissue regions.

## 4. Discussion

The principal contribution of this study is the integration of curved corneal geometry, epithelial, stromal, and endothelial cellular compartments, peripheral neural elements, apical ALI culture, and basal perfusion within a recoverable tissue construct. Previous models have separately introduced controlled corneal curvature [15], innervated three-dimensional epithelial-stromal tissue [16], or full-thickness epithelial-stromal-endothelial organization in compressed collagen [30]. The CMCoC brings these features together in a two-component PDMS device with circumferential wells for hiPSC-derived DRGOs. The tissue could be removed while retaining a visible curved profile, enabling both whole-mount and cross-sectional analyses. The contribution therefore lies in an integrated and experimentally accessible architecture rather than complete replication of the native cornea.

The curvature dome had a radius of 7.8 mm, selected to approximate a representative central anterior human corneal radius [14]. This value is a design reference rather than a universal anatomical measurement because corneal geometry varies among individuals and across the ocular surface. Incorporating curvature nevertheless moves the cellular environment beyond conventional planar culture, and organ-chip evidence indicates that curvature can modulate corneal stromal-cell behavior [15]. Curvature was combined with an apical ALI and continuous basal perfusion to establish distinct exposure compartments. In a cell-free 7.6-mm-radius dome model, thin-film behavior reflected coupled drainage, evaporation, and composition-dependent dewetting [36]. This observation motivates direct measurement of topical-fluid spreading, thinning, residence, and epithelial exposure on the accessible CMCoC apical surface in future dosing studies [13,23].

The CSC-laden plastically compressed collagen membrane provided a thin, manipulable stromal scaffold on which epithelial and HCEC-B4G12-containing surfaces could be established [30]. Whole-mount, orthogonal, and cross-sectional immunofluorescence imaging showed spatially distinguishable epithelial, stromal, and endothelial cellular compartments consistent with the intended arrangement. The epithelium was multilayered but thinner than the mouse corneal reference. A recent engineered epithelium-stroma substitute likewise formed multiple epithelial strata while retaining an incompletely developed basement membrane and reduced stromal extracellular-matrix components relative to native tissue [37]. This comparison distinguishes epithelial stratification and gross organization from complete maturation. The additional staining extends this cellular characterization: Na^+^/K^+^-ATPase was detected in the endpoint B4G12-containing layer, ALDH3A1 labeling was observed in epithelial and stromal regions, and no conspicuous α-SMA labeling was seen in the examined stromal regions. Lower corneal differentiation-marker expression has been reported in HCE-T relative to primary differentiated epithelium [38], providing context for the endpoint CK3 pattern. The present findings therefore identify the CMCoC as an organized multilayered tissue providing a structural basis for future investigations of interactions across epithelial, stromal, and endothelial cellular compartments.

A defining feature of the CMCoC was the circumferential placement of hiPSC-derived DRGOs. Native corneal nerves enter from the periphery, branch within the tissue, and extend toward the epithelium, where neural-epithelial interactions contribute to ocular-surface homeostasis [5,6]. Peripheral DRGO placement provided a spatially relevant origin for neurite extension toward the central construct, and βIII-tubulin-positive processes subsequently formed an extensive network associated with the epithelial compartment. Bioinspired anesthetic delivery and tissue-repair strategies provide relevant formulation context for future localized-delivery studies in this model [39]. This configuration combines concepts from innervated three-dimensional corneal tissue and compartmentalized neural microfluidics [16,17]. In mouse and primary limbal-cell systems, trigeminal nerve-derived substance P regulated limbal stem-cell behavior through NK1R/PI3K-AKT signaling [40], suggesting a candidate neuroepithelial mechanism for future testing in the CMCoC rather than a pathway established here. Patch-clamp comparison of adult human DRG neurons and hiPSC-derived sensory neurons also identified differences in rheobase, capacitance, firing behavior, and action-potential properties [41]. Together, the current βIII-tubulin findings provide a structural basis for subsequent stimulus-response, electrophysiological, and neuropeptide-signaling studies.

Morphometric analysis identified a higher βIII-tubulin-positive area fraction in CMCoC after correction for multiple comparisons, while the mean branch-point density showed a numerical increase. The native reference provides context for tissue layering and nerve-network organization. Human donor corneal anatomy further documents the progression from stromal nerve bundles through subepithelial and subbasal networks to epithelial terminals [42]. Relevant engineered configurations include primary human corneal cells combined with embryonic chick DRGs [16], and HCE-T/B4G12 cells combined with human stromal cells and hiPSC-derived DRGOs [30]. These studies provide complementary anatomical and tissue-engineering context for the CMCoC. Differences in epithelial thickness also need to be interpreted in the context of the immortalized HCE-T cell source and its culture-dependent differentiation state. The neural comparison involves developing hiPSC-DRGO-derived neurites and native corneal nerves of trigeminal origin [5,6]. In the CMCoC, ten peripheral DRGOs and medium containing 10 ng/mL NGF create a defined outgrowth configuration that may influence neurite coverage and branching. The higher values therefore describe the structures captured in the analyzed image regions, rather than greater physiological innervation. Human literature measurements are not treated as matched numerical benchmarks because acquisition depth, sampled region, and metric definitions vary across studies.

ZO-1 and F-actin delineated cellular organization without establishing barrier or transport function [43]. Application-specific use will require assessment of epithelial barrier integrity, endothelial transport, neural responsiveness, and optical performance. The photographs document a recovered curved profile but do not quantify its curvature or stability. Dedicated comparisons are needed to distinguish the contributions of geometry, perfusion, ALI, NGF, and DRGO co-culture. Organoid variability, matrix organization, cross-batch reproducibility, and device sealing and flow behavior are further characterization priorities. These considerations define the next steps toward fit-for-purpose qualification of the model [44] and its development for studies of corneal tissue interactions and treatment responses.

## 5. Conclusions

The CMCoC integrates a recoverable curved tissue with epithelial, stromal, and endothelial cellular compartments, circumferential DRGOs, apical ALI culture, and basal perfusion. The retrieved tissue showed a visible curved profile, the intended multilayer cellular arrangement, and an extensive βIII-tubulin-positive DRGO-derived neurite network associated with the epithelial compartment. These findings demonstrate the structural feasibility of combining these features within a recoverable corneal model and provide a basis for its further development.

## Data Availability

All STL files for 3D-printed molds are included in the Supplementary Materials. All other relevant data are available from the corresponding author upon reasonable request.

## Supplementary Materials

The following supporting information can be downloaded at https://www.mdpi.com/article/10.3390/biomedicines14102242/s1, Figure S1: DRGO differentiation protocol and immunophenotypic characterization; Figure S2: Epithelial and endothelial marker staining at the endpoint of CMCoC culture; Table S1: Summary of neural morphometric measurements in CMCoC and mouse cornea; Table S2: Individual source values for epithelial thickness and neural morphometry.

## Abbreviations

The following abbreviations are used in this manuscript:

3D	three-dimensional
3Rs	Replacement, Reduction, and Refinement
ALDH3A1	aldehyde dehydrogenase 3 family member A1
ALI	air–liquid interface
ARVO	Association for Research in Vision and Ophthalmology
CEnC(s)	corneal endothelial cell(s)
CEpC(s)	corneal epithelial cell(s)
CMCoC	curved multilayer cornea-on-a-chip
CSC(s)	corneal stromal cell(s)
DAPI	4′,6-diamidino-2-phenylindole
DMEM	Dulbecco’s modified Eagle medium
DMEM/F12	Dulbecco’s modified Eagle medium/nutrient mixture F-12
DRG	dorsal root ganglion
DRGO(s)	dorsal root ganglion organoid(s)
EDTA	ethylenediaminetetraacetic acid
F-actin	filamentous actin
HCE-T	human corneal epithelial cell line HCE-T
HCEC-B4G12	human corneal endothelial cell line B4G12
hiPSC(s)	human induced pluripotent stem cell(s)
LCD	liquid crystal display
NGF	nerve growth factor
NK1R	neurokinin 1 receptor
PBS	phosphate-buffered saline
PDMS	polydimethylsiloxane
PI3K–AKT	phosphoinositide 3-kinase–protein kinase B signaling pathway
SD	standard deviation
SMILE	small-incision lenticule extraction
STL	stereolithography file format
ZO-1	zonula occludens-1
