# Supplementary material for: A Recoverable Curved Multilayer Cornea-on-a-Chip with DRGO-Derived Neurites
Source: Biomedicines. 2026 Oct 3;14(10):2242. doi: 10.3390/biomedicines14102242 (PMC13701655; doi:10.3390/biomedicines14102242)
Supplement: Supplementary file 1 [file biomedicines-14-02242-s001.zip › Supplementary Materials.pdf]

Supplementary Materials

A Recoverable Curved Multilayer Cornea-on-a-Chip with DRGO-Derived Neurites

Xi Wang, Jianing Gu, Chengcheng Ding, Jiansu Chen, Zekai Cui

Figure S1. DRGO differentiation protocol and immunophenotypic characterization.
Figure S2. Epithelial and endothelial marker staining at the endpoint of CMCoC culture.
Table S1. Summary of neural morphometric measurements in CMCoC and mouse cornea.
Table S2. Individual source values for epithelial thickness and neural morphometry.

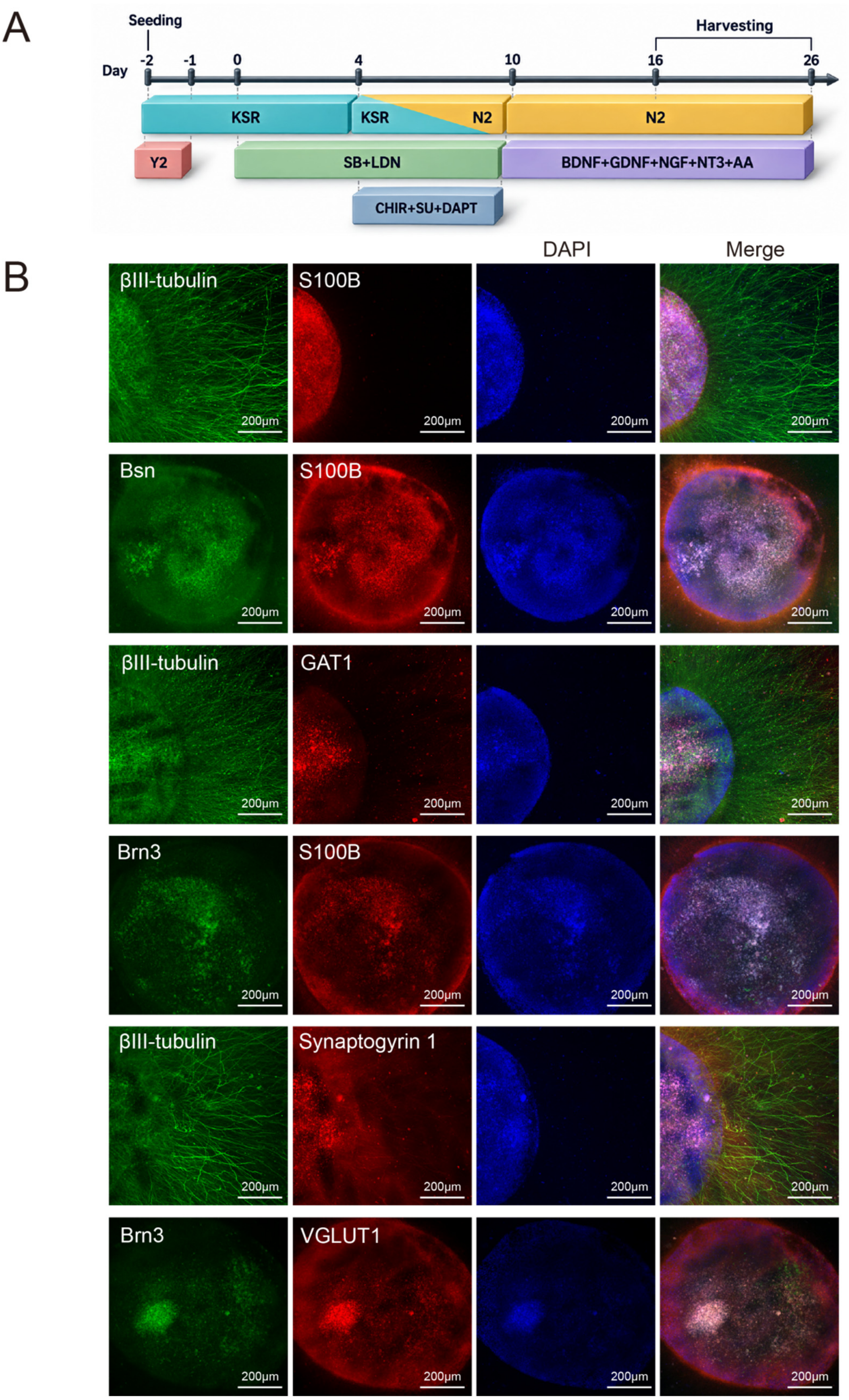

Figure S1. DRGO differentiation protocol and immunophenotypic characterization.
(A) Schematic of the differentiation protocol. Cells were seeded on day −2, and DRGOs were

collected between days 16 and 26. KSR-based medium was progressively replaced by N2 medium between days 4 and 10. Factor-supplementation intervals are indicated along the timeline. Abbreviations: DRGO, dorsal root ganglion organoid; KSR, knockout serum replacement; Y2, Y-27632; SB, SB431542; LDN, LDN193189; CHIR, CHIR99021; SU, SU5402; BDNF, brain-derived neurotrophic factor; GDNF, glial cell line-derived neurotrophic factor; NGF, nerve growth factor; NT3, neurotrophin-3; AA, ascorbic acid. (B) Representative immunofluorescence images of differentiation-day-26 DRGOs before chip incorporation. From top to bottom, the marker pairs are βIII-tubulin/S100B, Bassoon (Bsn)/S100B, βIII-tubulin/GAT1, Brn3/S100B, βIII-tubulin/Synaptogyrin 1, and Brn3/VGLUT1. The first and second markers in each pair are shown in green and red, respectively. Nuclei are counterstained with DAPI (blue), and merged images are shown in the rightmost column. Scale bars: 200 μm.

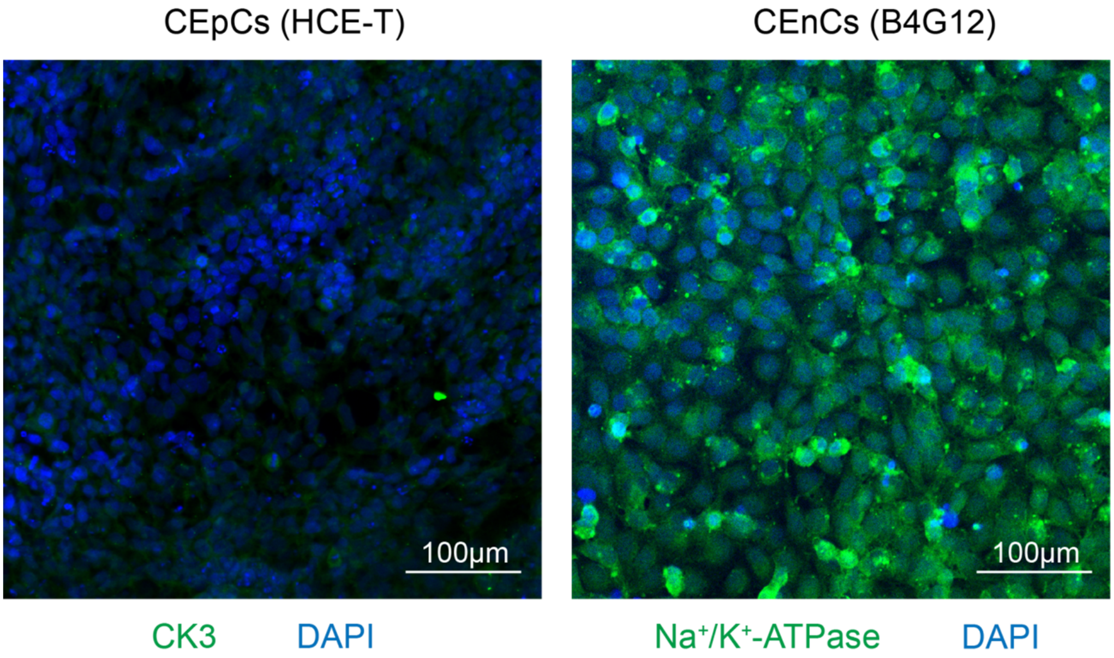

Figure S2. Epithelial and endothelial marker staining at the endpoint of CMCoC culture. Representative immunofluorescence images of the HCE-T epithelial compartment stained for CK3 (left) and the B4G12 endothelial compartment stained for Na^+/K^+-ATPase (right) at the chip-culture endpoint. Marker staining is shown in green, and nuclei are counterstained with DAPI (blue). Scale bars: 100 μm

Table S1. Summary of neural morphometric measurements in CMCoC and mouse cornea.

Outcome	CMCoC, mean ± SD	Mouse cornea, mean ± SD	CMCoC−mouse difference (unadjusted 95% CI)	Unadjusted P	Holm-adjusted P
Neurite area fraction (0–1)	0.2146 ± 0.0167	0.1736 ± 0.0110	0.04102 (0.01559 to 0.06644)	0.008626	0.034505
Structure length density (mm/mm^2)	17.92 ± 3.09	14.86 ± 2.35	3.06 (−1.77 to 7.89)	0.169747	0.339494
Branch-point density (mm^{-2})	811.38 ± 138.15	566.86 ± 102.77	244.53 (29.57 to 459.49)	0.032275	0.096824
Endpoint density (mm^{-2})	1504.67 ± 187.22	1454.65 ± 137.88	50.02 (−240.64 to 340.67)	0.683335	0.683335

Values are presented as mean ± SD from four specimens per group. Comparisons used two-sided Welch's t-tests, with Holm correction across the four outcomes. Confidence intervals are unadjusted; inference for the four neural comparisons is based on the Holm-adjusted P values.

Table S2. Individual source values for epithelial thickness and neural morphometry.

A. Epithelial thickness

Record	CMCoC (μm)	Mouse cornea (μm)
1	24.3	33.6
2	22.7	35.1
3	19.8	40.3
4	28.9	41.9
5	20.5	34.2

B. Neural morphometry

Source record	Area fraction	Length density (mm/mm^2)	Branch-point density (mm^{-2})	Endpoint density (mm^{-2})
CMCoC 1	0.228383	21.025303	922.528879	1706.122685
CMCoC 2	0.220735	16.441787	794.708612	1461.596958
CMCoC 3	0.219014	19.885020	905.856670	1583.859822
CMCoC 4	0.190316	14.320655	622.429123	1267.087857

Mouse 1	0.175498	16.885132	550.182885	1561.630210
Mouse 2	0.157542	11.945359	444.592231	1272.645260
Mouse 3	0.181729	16.634635	694.675360	1561.630210
Mouse 4	0.179604	13.982106	577.969900	1422.695138

Record numbers indicate the order of values within each source data series. Values in panel B are rounded to six decimal places for display; statistical calculations used the unrounded values.
